# Evaluation of Group Genetic Ancestry of Populations from Philadelphia and Dakar in the Context of Sex-Biased Admixture in the Americas

**DOI:** 10.1371/journal.pone.0007842

**Published:** 2009-11-25

**Authors:** Klara Stefflova, Matthew C. Dulik, Athma A. Pai, Amy H. Walker, Charnita M. Zeigler-Johnson, Serigne M. Gueye, Theodore G. Schurr, Timothy R. Rebbeck

**Affiliations:** 1 Department of Biostatistics and Epidemiology, School of Medicine, University of Pennsylvania, Philadelphia, Pennsylvania, United States of America; 2 Department of Anthropology, University of Pennsylvania, Philadelphia, Pennsylvania, United States of America; 3 Hôpital Général de Grand Yoff and Université Cheikh Anta Diop, Dakar, Senegal; 4 Abramson Cancer Center, School of Medicine, University of Pennsylvania, Philadelphia, Pennsylvania, United States of America; State University of New York College at Oneonta, United States of America

## Abstract

**Background:**

Population history can be reflected in group genetic ancestry, where genomic variation captured by the mitochondrial DNA (mtDNA) and non-recombining portion of the Y chromosome (NRY) can separate female- and male-specific admixture processes. Genetic ancestry may influence genetic association studies due to differences in individual admixture within recently admixed populations like African Americans.

**Principal Findings:**

We evaluated the genetic ancestry of Senegalese as well as European Americans and African Americans from Philadelphia. Senegalese mtDNA consisted of ∼12% U haplotypes (U6 and U5b1b haplotypes, common in North Africa) while the NRY haplotypes belonged solely to haplogroup E. In Philadelphia, we observed varying degrees of admixture. While African Americans have 9–10% mtDNAs and ∼31% NRYs of European origin, these results are not mirrored in the mtDNA/NRY pools of European Americans: they have less than 7% mtDNAs and less than 2% NRYs from non-European sources. Additionally, there is <2% Native American contribution to Philadelphian African American ancestry and the admixture from combined mtDNA/NRY estimates is consistent with the admixture derived from autosomal genetic data. To further dissect these estimates, we have analyzed our samples in the context of different demographic groups in the Americas.

**Conclusions:**

We found that sex-biased admixture in African-derived populations is present throughout the Americas, with continual influence of European males, while Native American females contribute mainly to populations of the Caribbean and South America. The high non-European female contribution to the pool of European-derived populations is consistently characteristic of Iberian colonization. These data suggest that genomic data correlate well with historical records of colonization in the Americas.

## Introduction

Populations of the present-day Americas were shaped by diverse incoming groups and their intermixing. Although the number of the Native Americans was greatly reduced due to conflict and disease they, together with the early arriving Europeans and surviving Africans brought to the Americas during the massive African Diaspora, all left their genetic imprint in multiple admixed populations. Later, several other immigrant groups from around the World (e.g. Asian populations) and increasingly common admixture among the existing groups further amplified the admixed character of this continent. This complicated ancestry and admixture is reflected in an individual's genetic background. While recognizing that each individual is genetically unique, it is still common in epidemiology to categorize people into a few self-identified races (SIRE) that partly reflect the complicated history of each group, yet fail to predict the *extent* of the contribution from each parental population to different SIRE groups [1].

One example where SIRE may inadequately represent this diverse contribution from parental populations is population stratification bias in molecular epidemiology case-control association studies [2]. While ancestry information in molecular epidemiology is usually studied in terms of individual ancestry, maternal and paternal group ancestry can provide information about the ancestry of populations without making inferences about individuals, accounting for all significantly contributing populations. Uniparentally inherited mitochondrial DNA (mtDNA) and Y-chromosome (NRY) behave as uninterrupted single loci that are often used in estimating group ancestry and predicting gender-specific population demographic processes [3], [4]. Together, they can accurately reflect the average autosomal group ancestry [5]. Individually, they can help to separate gender-biased admixture processes [6], [7].

Because they have a higher incidence of several common diseases [8] and a complicated history, African Americans have been studied in a variety of epidemiological and population genetic settings. As a SIRE group, they represent the descendants of Africans brought mainly during the African Diaspora from W/WC/SW/SE Africa, admixed with Europeans and possibly Native Americans. Still, every regional group of African Americans may have been drawn from different African sources or have a unique history that will influence the extent and pattern of admixture and make them a unique group. Similar regionally-specific admixture most likely influences other groups in the Americas.

Based on reports of low resolution maternal and paternal ancestry, mtDNA and NRY reveal a sex-biased gene flow from European males to US/Jamaican African Americans, but the extent of group maternal and paternal European admixture greatly varies [5], [9] (e.g. 0–15% for mtDNA and 8.6–46.9% for NRY in 9 US populations in Parra et al.). This sex-biased admixture was also reported in African-descended populations in Uruguay [10] and both White and African Brazilians [11]–[13], while a study dealing with the FBI mtDNA database shows limited gene flow of non-Europeans to the pool of US European Americans [14]. Detailed assessment of African, Native American, and European admixture in both European- and African-descended groups in different populations of the Americas may improve our understanding of variation within and between each SIRE group from different regions. This combined analysis can help to approximate the parental populations for any uncharacterized group in each region during early epidemiological study design and aid in understanding if African- and European-descended American SIRE means the same in different parts of the Americas.

Therefore, we estimated maternal and paternal continental admixture proportions and report on the group ancestry of three populations: admixed Philadelphian African Americans and European Americans, as well as a control sample of Senegalese from Dakar as one of the possible source populations. Further, we have mined the published literature for raw mtDNA/NRY data (Brazil and Cuba) or ancestry estimates (Caribbean, Colombia, Uruguay) as well as census data in order to interpret our results within the context of the Americas.

## Methods

### Sampling, DNA Handling

The Philadelphia samples consisted of 217 self-identified African Americans and 204 self-identified European Americans. These individuals were ascertained between 1995 and 2007 as part of a prostate cancer case-control study, with cases identified through Urologic Oncology Clinics at multiple hospitals of the University of Pennsylvania Health System (UPHS) and controls being men attending UPHS general medicine clinics. Additionally, 49 subjects from Senegal (all cancer-free controls) were identified and ascertained from university and hospital populations in Dakar, Senegal. All study subjects from US and Senegal provided written informed consent for participation in this research. IRB approval for this study has been provided by the Committee on Studies Involving Human Beings of the University of Pennsylvania (Protocol #3614-2) and by the Commission Ethique et Evaluation at the Hopital General de Grand Yoff in Dakar (FWA 00002772).

Genomic DNA was obtained from buccal swabs (Cyto-Pak Cytosoft Brush, Medical Packaging Corporation, Camarillo, CA) processed using either a protocol modified from Richards et al. [15] as described previously [16] or using a modified protocol on the Qiagen 9604B robot with the QIAamp 96 DNA Buccal Swab Biorobot Kit (Valencia, CA). Prior to typing, the whole genome of these samples was amplified using the GenomePlex Complete Whole Genome Amplification kit (Sigma, St. Louis, MO).

### mtDNA, NRY, and AIMs Typing

**Figure 1:**
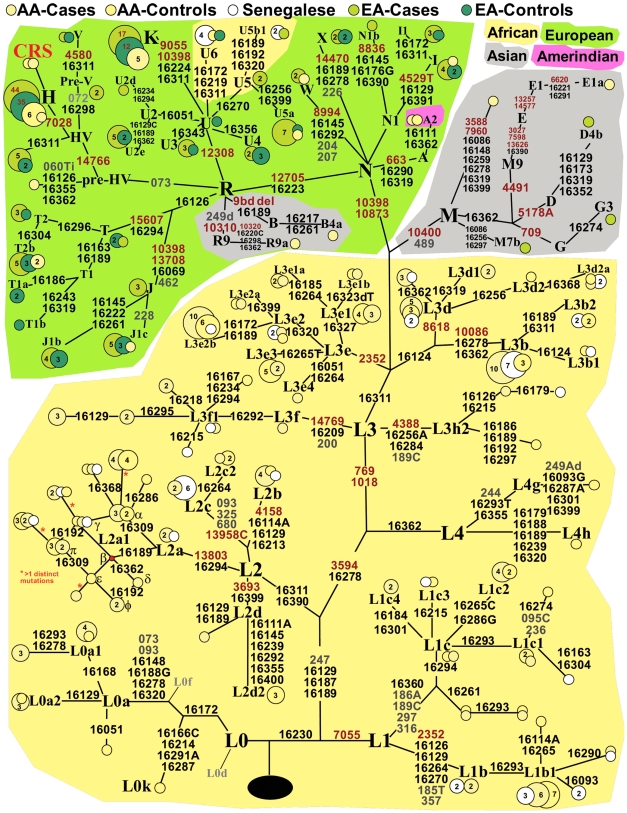
Mitochondrial DNA phylogenetic tree. Tree of mtDNA haplotypes based on median joining network with African American cases (yellow), African American controls (orange), Senegalese (white), European American cases (light green), and European American controls (dark green). Node sizes are proportional to the sample sizes, indicated by numbers within the node, with the exception of haplogroups H and K labeled by numbers in red. Variable positions typed for these samples in coding, HVS I, and HVS II region are distinguished by red, black and grey font, respectively. The main continental location is indicated by the background color with ochre indicating predominantly African, green West Eurasian, grey Asian, and pink Amerindian haplogroups. The raw data can be found in **File S1**.

The first and second hypervariable segments (HVS I and HVS II) of mtDNA were amplified and both strands sequenced between bp 16,030–16,490 and bp 50–710 (**Table S1**) using a BigDye™ Terminator v 3.1 (Applied Biosystems) after purifying with ExoSAP-IT (USB, Cleveland, OH). After purification with the QIAquick 96 PCR Purification kit (Qiagen), the sequences were read using an ABI Prism 3130*xl* Genetic Analyzer (Applied Biosystems) and analyzed using Sequencher v4.7 software (Gene Codes Corp., Ann Arbor, MI). Each sample was then hierarchically typed for mutations in the mtDNA coding region using Restriction Fragment Length Polymorphism (RFLP) assays to correctly assign each of them into a particular haplogroup (**Table S2**). A phylogenetic tree (**Figure 1**) was drawn manually, based on the median-joining tree constructed using Network 4.5.0.2 [17], listing the diagnostic mutations RFLP-typed in coding and present in sequenced hypervariable regions.

**Figure 2:**
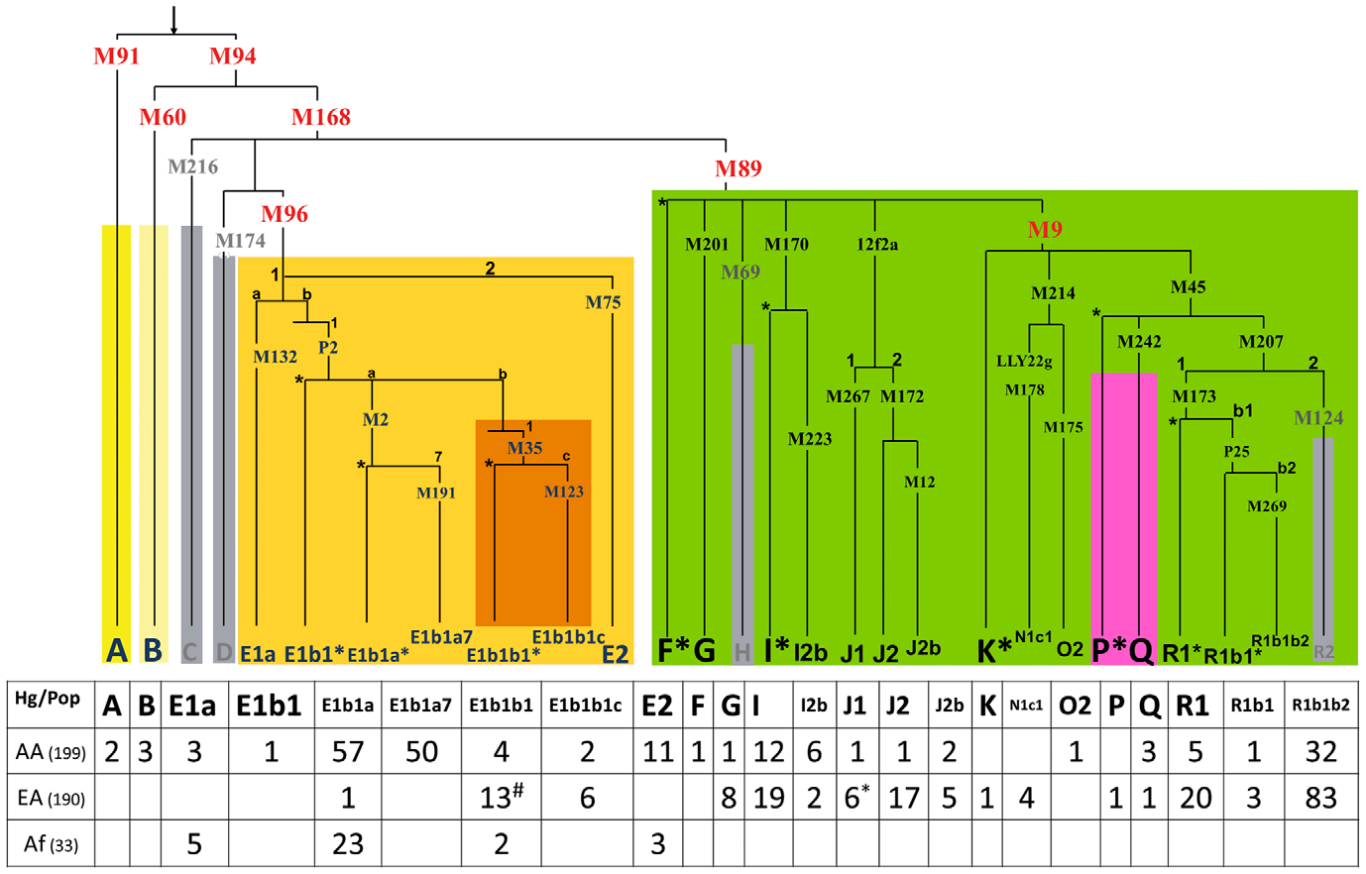
Y chromosome phylogenetic tree. NRY tree haplogroups observed in Philadelphia and Senegal data with typed SNPs indicated on each branch. Associated with each branch is the number of samples observed for each haplogroup in the pool of African Americans (AA, n = 199), European Americans (EA, n = 190), and Senegalese (Af, n = 33). We have omitted from the NRY tree M148 SNP designating E1b1b1a3a (formerly E3b1a). One EA sample belonging to this haplogroup was added to E1b1b1 (#). One EA sample was 12f2a* (Hg J) but was grouped with J1 (*) because of space constraints. The raw data can be found in **File S1**.

For NRY, the samples were typed using pre-designed TaqMan assays in combination with multiplex fragment analysis and RFLP (**Figure 2**). The SNPs are listed in **Table S3** (see also **References S1**) together with the haplogroup designation established by the Y-Chromosome Consortium in 2002 [18] and revised in Karafet et al. 2008 [19]. Both mtDNA, NRY variation and ethnicity information is listed in **File S1**.

For a small subset of the reported samples (31 African Americans, and 6 European Americans), we also estimated the autosomal ancestry by genotyping the samples on a commercially available Illumina Golden Gate 1509 AIMs chip. The resulting genotypes were combined with available genotypes from an Illumina admixture panel (YOR, CEU, JPT+CHB) that represent the ancestral African, European and Asian populations. The corresponding admixture proportions were estimated using the program STRUCTURE running 10,000/50,000 burn-in/repetitions, assuming an admixture model with correlated allele frequencies, running K = 1–5 and reporting estimates for K = 3 founding populations, where the posterior probability ln P(D) plateaus (**Table S4**).

### Phylogenetic and Statistical Analysis

**Figure 3:**
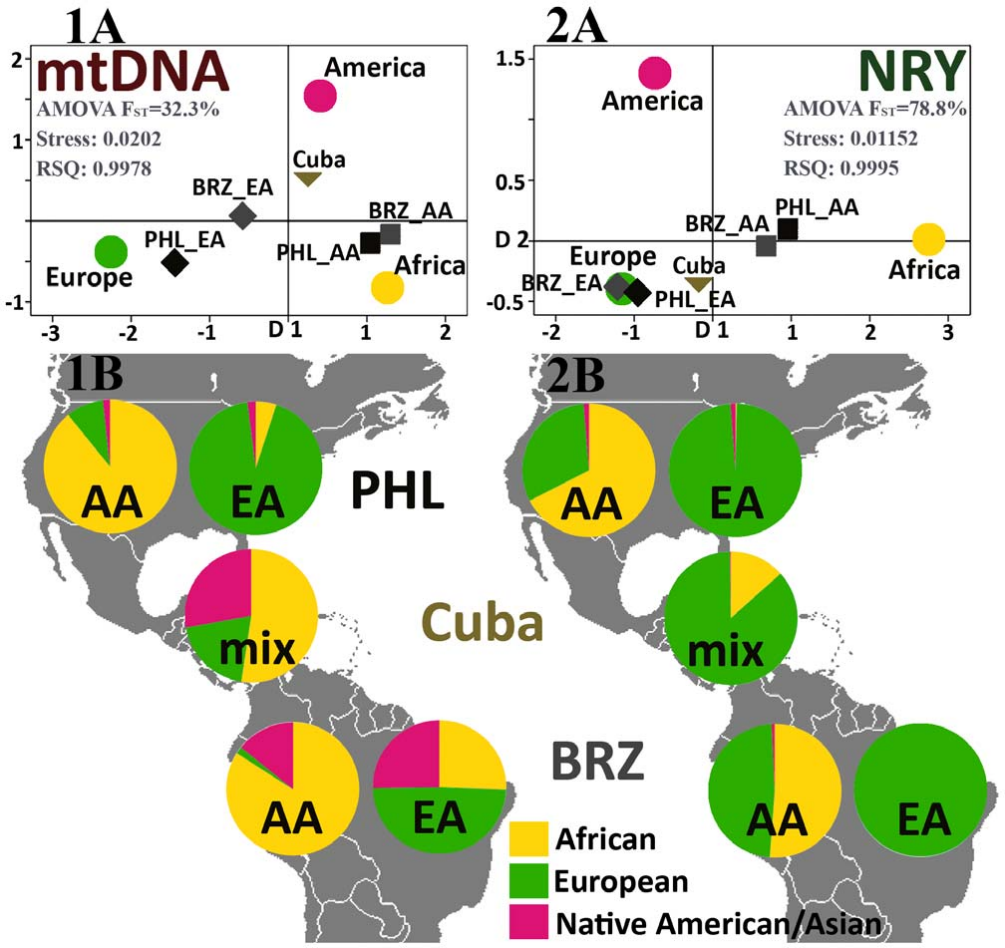
Contribution of European, African, and Native American female and male lineages to the populations of Philadelphia, Brazil, and Cuba. MtDNA (**1**) and NRY (**2**) profile of African Americans or African Brazilians (AA) and European Americans or White Brazilians (EA) from USA (Philadelphia, PHL) and Brazil (BRZ), as well as general the population of Cuba (mix) projected either (**A**) as their position in the multidimensional scale plots (MDS) depicting the genetic distances with respect to Africa, Europe, and America projected onto the two dimensional plane or (**B**) as pie charts, showing the relative contributions from African (yellow), European (green) and Native American/Asian (pink) populations calculated by ADMIX. These complementary analyses show gender-biased admixture in South and North Americans of primarily African or European descent. AMOVA F_ST_ represents the variation captured between the three parental continents.

We used Arlequin 3.11 [20] to estimate genetic distances based on Slatkin's linearized F_ST_ to construct multidimensional scaling (MDS) plots to assess “between group”, “within-population” and “between population within group” variation *via* the analysis of molecular variance (AMOVA) [21]. We included the phylogenetic relationship of mtDNA haplotypes/NRY haplogroups in the form of haplotypes (mtDNA) or distance matrix (NRY) and assumed Tamura and Nei's [22] model for nucleotide substitution for mtDNA sequences. The MDS plots were constructed using SPSS with input data in the form of an Arlequin-generated matrix of Slatkin's linearized F_ST_ distances [23]. For each MDS plot, we report the stress and RSQ statistics, which summarize the goodness of fit of multidimensional data in two dimensions. Additionally, AMOVA was reported for the parental populations (indicated in each MDS plot in **Figure 3**) showing the percentage of variation captured by defining the continental groups.

### Admixture Analysis

Admixture was estimated using the ADMIX 2.0 [24] software. We ran 50,000/100,000 (mtDNA/NRY) bootstrap simulations and report the estimates as a percent of contribution from a particular parental population, along with an estimate of the sampling error (SD). These calculations incorporate molecular divergence and haplotype frequencies, with both mtDNA and NRY being treated as a single locus. We initially explored K = 2–4 of founding populations and both based on the SD of the estimates and previous reports, we only pursued K = 3 where the Native American and Asian founding populations seem to be combined. For the mtDNA analysis, we used previously published sets drawn from: West Africa (n = 819, represented by Guinea Bissau [25], Senegal (our and published [26] data), and Sierra Leone [27]); Europe [28] (n = 3532); and the Americas [29] (n = 58) as the parental populations (note that the estimate represented by the Americas will partially overlap with the possible admixture from SE Asia). For the NRY analysis, our parental populations were defined as follows: West/West-Central Africa (n = 834, represented by Guinea Bissau [30], Mali, Ghana, Benin, Senegambia [7] (adding Senegalese reported in this publication) and Cameroon [7], [31]); West Eurasia [32] (n = 481, represented by Germany, Denmark, Galicia, and Turkey); and the Americas [33] (n = 398). The mtDNA and NRY variation in Brazilian admixed populations was first mined from the literature: Afro-Brazilian mtDNA [34]–[36] (n = 277) and NRY [12], [34], [35] (n = 380) and White Brazilian mtDNA [11], [12] (n = 247) and NRY [12] (n = 180) and mtDNA/NRY n = 245/132 of the general population of Cuba [37]. The combined datasets for each marker and group were subsequently analyzed for admixture using ADMIX. The genetic variation combined with phylogenetic distances was captured by 9–18 NRY haplogroups and 335 mtDNA haplotypes, defined by distance matrix (NRY) and sequences of HVS I/II and part of HVS III/coding region (mtDNA).

## Results

First, we deeply typed both mtDNA and NRY in Philadelphian African Americans and European Americans. We also typed the same markers in a group of Senegalese in order to gain insight into the detailed composition of one of the founding populations (e.g. the presence of “Eurasian” haplotypes) instead of relying on less well characterized published data. We then proceeded to phylogenetically analyze these haplotypes in parallel with admixture analysis using ADMIX, exploring the possible founding populations. Based on these analyses, we focused further on analysis using three founding populations (K = 3 was also corroborated by our STRUCTURE analysis using autosomal AIMs).

### Mitochondrial DNA (mtDNA)

We assessed the contribution of European, African and Native American populations to the pool of Senegalese, Philadelphian African Americans and Philadelphian European Americans by two means: 1) by admixture analysis using ADMIX [24] (deriving the possible parental (ancestral) regions from published sets comprising West/West Central Africa, Europe/West Eurasia, and the Americas), and 2) by counting haplotypes assigned to be of West Eurasian, Native American, Southeast Asian and African origin based on published literature and deep phylogenetic analysis of our samples. The mtDNA variation in all three populations sampled is shown in **Figure 1** using a phylogenetic tree, adapted from its median-joining outline [17].

Among 49 Senegalese, ancestry was composed mainly of African haplogroups, except for six individuals with haplotypes of Eurasian origin that are commonly found in North Africa (U6 and U5b1b [38], [39], **Table 1**). The ancestral composition of 217 Philadelphian African Americans was estimated by ADMIX to be 9.1% European (SD: 3%), 1.7% Native American (SD: 0.9%), and 89.2% African (SD: 3%). Additional details of this distribution can be found in **Table S5** (see also **References S1**). The breakdown of major mtDNA haplogroups and haplotypes is presented in **Table 1**. These data suggest that our admixture estimates are almost identical to the ancestry frequencies based simply on counting the known West Eurasian/Native American/SE Asian/African haplotypes. From these estimates, the ancestral contributions were counted to be 10.1% West Eurasian, 1.4% Native American and 87.1% African. In addition, 1.4% of ancestry was comprised of other haplogroups, including R9a (East Asian), E1a (Melanesian) and a haplogroup M sequence of unknown origin.

**Table 1:** Mitochondrial DNA variation in the Senegalese and Philadelphian populations.

Geogr. origin	Major haplogr.	Sub-haplogr.	AA Controls n = 90	AA Cases n = 127	Senegal Controls n = 49	EA Controls n = 78	EA Cases n = 126
America	A/B	A2/B4	1/0	1/1	-	-	-
Asia	R/E/M	R9/E1/M	-	1/1/1	-	-	-
	G/N/D/M	G/N1b/D4/M7b	-	-	-	-	1/1/1/1
W Eurasia	H/pre-HV	H/pre-HV	7/0	2/1	-	35	44
	HV/V	HV/V	-	-	-	2/1	5/3
	J/T	J/T	0/1	1/2	-	6/8	12/9
	U	U/K	1/0	0/5	-	8/12	20/17
	I/W/X	I/W/X	-	1/1/0	-	3/0/2	7/2/2
Africa	U6/U5*	U6/U5b1	1/0	-	4/2	1/0	0/1
	L0	L0a/L0k	3/1	10/0	-	-	-
	L1	L1b/L1c	8/9	9/10	8/2	-	-
	L2	L2a	17	21	5	-	-
		L2b	1	4	1	-	-
		L2c	2	3	6	-	-
		L2d	3	1	-	-	-
	L3	L3b	6	10	10	-	-
		L3d	5	11	5	-	-
		L3e	20	20	3	-	-
		L3f/L3h	1/1	9/1	2/1	-	-
	L4	L4h/L4g	1/1	-	-	-	-

Major mtDNA haplogroups present in Philadelphian African American (AA), European American (EA), and Senegalese samples. The phylogenetic details are depicted in the tree in **Figure 1** and the sequences presented in **File S1**.

(*Note: Although haplogroup U is of Eurasian origin, we placed the U6 and U5b1 haplotypes under “Africa” since they are commonly found in North African populations).

The ancestral composition of 204 Philadelphian European Americans was estimated to be 93% European (SD: 7%), with a small (although not significant) contribution from Native American (1.6%, SD: 2%) and African (5.5%, SD: 5%) populations (**Table S5**). Further analysis of these mtDNA haplotypes confirmed that the ancestry of nearly all Philadelphian European Americans is of European origin. We observed mainly West Eurasian haplotypes (**Table 1**), with the exception of five haplotypes that can be considered East Asian (G3, M7b, and D4 [40]) and North African (U6), accounting for the non-zero ADMIX estimates of Native American (here overlapping with SE Asia) and African admixture in this population.

### Y Chromosome (NRY)

The ancestry of 33 Senegalese individuals was composed solely of African haplogroup E, reflecting the typical pool of West African NRY chromosomes [30]. The ancestral composition of 199 Philadelphia African Americans, based on admixture analysis estimates, was 31.2% West Eurasian (SD: 4%), 1.3% Native American (SD: 1.5%), and 67.5% African (SD: 4%) (**Table S5**). For the NRY haplogroups, summarized in **Figure 2**, we see nearly identical estimates: 31.5% West Eurasian, 1.5% Native American, and 67% African.

In contrast, the ancestral composition of 190 Philadelphia European Americans was estimated by admixture analysis to be almost 100% European (98.3% West Eurasian (SD: 3%), 1.1% Native American (SD: 1.5%), and 0.6% African (SD: 1.4%)). These estimates were consistent with analysis of continent-specific NRY haplogroups: 98.5% West Eurasian, 1% Native American and 0.5% African haplogroups. The NRY variation set into a phylogenetic tree is depicted in **Figure 2**.

### Autosomal Ancestry Informative Markers (AIMs)

In addition to the maternal and paternal ancestry, we have assessed the autosomal ancestry for a subset of African American (n = 31) and European American (n = 6) samples by genotyping these on the commercially available Illumina 1509 AIMs chip, followed by estimating the admixture proportions using the program STRUCTURE (K = 3) (**Table S4**). These estimates show >20% European ancestry in African Americans (23.7%) and a small African component in European Americans (2.5%) with Native American/SE Asian populations contributing less than 2% to both. These estimates were compared to the putative autosomal group ancestry proportions calculated from the maternal and paternal admixture estimates.

### Gender-Biased Admixture in the Americas

We compared admixture patterns in two populations self-identified as either primarily of African or European ancestry from South America (Brazil) and North America (Philadelphia). As shown in **Figure 3**, we present Multidimensional Scaling (MDS) plots (based on Arlequin-derived Slatkin's F_ST_ genetic distances [20]) and admixture estimates depicted as pie charts. These admixture estimates were calculated using ADMIX [24], defining the three possible ancestral regions as West/West Central Africa, Europe/West Eurasia, and the Americas (and SE Asia, in our initial analysis considering four populations) and using phylogenetic relationships between the observed haplotypes. We observed a striking difference in the extent of admixture between North and South America in both populations, with Brazilians having in general higher admixture with the exception of White Brazilian NRYs. Additionally, NRY and mtDNA profiles, reflecting gender-specific admixture patterns, suggest diverging patterns of admixture in male and female populations. This gender-biased admixture is clearly identifiable both by the position in MDS plots and ancestry proportions of the general population of Cuba (**Figure 3**). These data suggest that it is primarily European males and African/Native American females that contributed to the genetic ancestry of admixed populations of the Caribbean/South America.

## Discussion

We have characterized the mitochondrial DNA (mtDNA) and non-recombining portion of Y-chromosome (NRY) variation in a sample from Senegal as well as two major groups of Philadelphians: self-identified European Americans and African Americans. These two groups comprise over 88% of the Philadelphian population (45% and 43.2%, respectively, according to the 2000 U.S. Census). We found mainly African haplogroups in the Senegalese sample, with the exception of 12.2% of Senegalese (3 Wolof, 2 Fulbe, and 1 Sahalle) carrying U6 and U5b1b mtDNA haplogroups that, although haplogroup U is of Eurasian origin, can be found throughout North Africa as a result of an ancient migration back to Africa. In Philadelphian African Americans, we observed a significant European admixture (mtDNA>9% and NRY>31%) as well as a small (<2%) contribution from Native Americans. To calculate the corresponding autosomal ancestry of self-identified African Americans, accounting for both maternal and paternal contributions, we used our data to compute m_AUTO_ = ½ m_mtDNA_ + ½ m_NRY_ [5], which was estimated to be: 78.4% African, 20.1% European, and 1.5% Native American. These calculated estimates seem to accurately reflect the autosomal group admixture, based on typing a small subset of samples using autosomal AIMs (n = 31, 74.4% African, 23.7% European, and 1.9% SE Asian/Native American, **Table S4**). Also, these estimates parallel previous reports, although our estimates suggest a higher European contribution, especially compared to the 12.7–13.8% autosomal and 2.8–11% low resolution maternal European ancestry found in a sample from Philadelphia reported by Parra et al. [9]. For example, European contribution to NRY, autosomes, and mtDNA was estimated to be 28.46%, 19.99%, and 8.51%, respectively, in African Americans from Pittsburg, Chicago, Baltimore and North Carolina [5], or autosomal ancestry of African Americans from NY state was estimated to be 83% African, 15% European and 2% Native American [1].

In contrast to the admixed nature of African Americans, we observed little admixture in the European American sample (<7% in mtDNA and <1.7% in NRY). Group ancestry or uniparental admixture in European Americans has not been widely reported. However, reports of admixture using the FBI mtDNA database [14] or autosomal loci have presented estimates that are consistent with our findings that European populations have contributed the vast majority of ancestry of European Americans. In our case, the calculated autosomal admixture is 95.8% European, with African and Native American contributing less than 5% (2.8% African, 1.4% SE Asian/Native American), consistent with published work (1.6% and 1.2%, respectively, in the US [41]), as well as our own autosomal estimates from a subset of the samples (95.7% European).

To further characterize the ancestry of Philadelphian populations within the global context, we mined the literature for published reports of mtDNA and NRY variation, selecting Brazil and Cuba as representative of South America and Caribbean that have sufficient resolution, sampling range, and sample size to represent the country. First, we analyzed admixture in published reports that contained mtDNA and NRY data from White and African Brazilians that were comparable to the data we collected in our Philadelphia sample (**Figure 3**). This analysis revealed directional admixture patterns. First, separating the maternal and paternal admixture shows clearly that European males contributed to the populations of America to a greater degree than European females. This is true for both African- and European-derived Americans, although less pronounced in the case of the Philadelphian European American sample. The admixture data in the general population of Cuba support this trend.

Therefore, while male admixture is dominated by European Y-chromosomes, the female admixture shows a remarkable influence of African and Native American female ancestors, the latter prominent mainly in the South American/Caribbean pool, as seen in Brazilians and Cubans. For example, both African Americans and African Brazilians have a high percentage of admixture from European NRYs and some non-African mtDNA admixture that is drawn mainly from European or Native American mtDNA pools in North and South America, respectively. On the other hand, both European Americans from Philadelphia and White Brazilians [11] do not show admixture in their paternal gene pool (NRY being almost 100% European in both cases), while, as in the case of African-derived populations, the African and Native American mtDNAs contribute greatly to the maternal pool of White Brazilians. This is in contrast to the maternal pool of Philadelphian European Americans that shows <7% admixture from non-European sources, consistent with the European Americans from the FBI mtDNA database [14]. Thus, there are distinct differences between North and South America in the extent of admixture from the three founding populations in the pool of New World individuals who self-identify as “black” and “white” (**Table S5**).

To further investigate whether the patterns we observed in Philadelphian, Brazilian, and Cuban populations have a similar impact in other countries in the Americas, we compared published mtDNA and NRY frequencies of African-derived and general populations, considering the demographics of the investigated countries. Focusing first on the published mtDNA and NRY admixture of African-derived populations of the Caribbean, Colombia, Brazil, and Uruguay, it is clear that they show the same trend as the African American and African Brazilian populations analyzed in this paper (**Table 2**). Namely, from North to South, there is a decrease in the contribution of both maternal and paternal African ancestry, mainly due to admixture with Native American females and European males. Also, we detect the same sex-biased admixture, where more African females than males contributed to the pool of African-derived populations across the Americas. While the observed North-South trends seem to be consistent in African American populations, in order to dissect in greater depth the processes that shaped the populations of North and South America differently, we turned our focus to European-derived populations.

**Table 2:** Published mtDNA and NRY profiles of African-descended Americans.

Africa-derived populations	mtDNA			NRY			Citations
	Africa	Eurasia	America	Africa	Eurasia	America	
**USA**	**94.7%**	3.7%	**1.6%**	**73.6%**	**26.4%**	0%	[33], [49]
Caribbean*	**90.3%**	4.3%	**5.3%**	**68%**	**32%**	0%	[50]
**Colombia**	**78.8%**	1.6%	**19.7%**	**63%**	**36%**	1%	[51], [52]
**Brazil**	**76.7%**	3%	**20.3%**	**52.7%**	**44.3%**	3%	[12], [13], [34]–[36], [53], [54]
**Uruguay**	**52.3%**	19%	**28.7%**	**30.2%**	**64.1%**	5.1%	[10]

The bold numbers indicate the North-South gradient of decreasing or increasing trends in African and Native American/West Eurasian admixture for mtDNA and NRY.

(*Note: Afro-Caribbeans represent the populations from the islands of Dominica, Grenada, Jamaica, St. Kitts, St. Lucia, St. Thomas, St. Vincent, and Trinidad).

Since previously published data on maternal and paternal ancestry of European-derived populations are scarce [11], [14], we studied this group indirectly by correlating the known mtDNA and NRY ancestry of African-derived and general populations with demographic information. First, although only 5% of the African slave trade arrived in North America [42], the US has the highest proportion of self-identified African Americans (∼13%) out of the regions studied (with the exception of some of the Caribbean islands, such as Jamaica, which has up to 91% self-identified African-descended individuals). This implies that a significant portion of African parental variation in South America (and parts of the Caribbean) exists as part of admixed populations (e.g., Mulato and Pardo, although little African admixture was reported in Mestizos [43]). To evaluate whether the genetic data are consistent with this hypothesis, we calculated the proportion of African male and female lineages that were contributed to the general population *solely* by individuals who self-identify as “black” or African (**Table S6**). Nearly all of African Y-chromosomes are found in individuals that self-identify as “black”, whereas less than 50% of the African mtDNAs are found in these individuals. In other words, a significant fraction of African mtDNAs are found in groups that do not self-identify as “black”. To determine which other populations these African maternal lineages significantly contribute to, we estimated the possible admixture in European-derived populations. While these estimates are only approximate, the proportion of contribution of European females to the pool of “white” individuals in the Iberian-founded Caribbean and South America is clearly lower than to European Americans in the United States, with variable proportions of African and Native American females contributing to each of these populations (**Table S7**).

Sex-biased admixture is not a process unique to the Americas. The pattern of NRY variation documents this phenomenon on every continent. For example, the unique Y chromosome lineage spread by (males related to) Genghis Khan over the vast steppes of Asia [44] or uni-directional mating of Bantu males and Pygmy females [45]–[47] can both serve as examples of the history of a population being reflected in Y chromosome phylogeography in the Asian and African continents. In the Americas, European males contributed significantly to all admixed populations [43]. However, the difference between North and Caribbean/South America lies both in the diverse cultural histories that categorized people of admixed ancestry either by descent or color [48], as well as the availability of European women. While individuals with any amount of African ancestry were considered “African American” in the United States (the “one drop rule”), in Brazil, where most of the first settlers were male, unions between European males and Native American/African females were common and the “skin tone” of offspring was used to define an individual's “race” [14], [36]. Therefore, in contrast to European North Americans, who have relatively low levels of non-European admixture from both male and female predecessors, individuals categorized as “White” Brazilians show higher levels of African and Native American admixture. This non-European ancestry is almost entirely derived from maternal lineages.

There are several limitations of our study. First, our estimates of admixture in European-derived populations in the Americas should serve only as approximations, since this information was mined from indirect sources (genetic data from complementary populations and demographics). Second, in spite of the advantages of using uninterrupted single locus-like information to trace maternal and paternal ancestry, the use of uniparental markers is limited to group ancestry estimates, bearing only very limited information about the ancestry of an individual. Care should therefore be exercised when interpreting our results on anything other than the group ancestry level.

We have shown that estimates of group ancestry derived from combined mtDNA and NRY admixture estimates predict average autosomal ancestry. When separated, these estimates mirror gender-specific admixture processes, reflecting diverse socio-historical demographic processes. Also, groups sharing the label of self-identified race across the Americas are often shaped by different social pressures and this will be reflected in their genome. This may add to the complexity of the population stratification issue in molecular epidemiology, which strives for enhancing the analysis by increasing the number of individuals. In the future, characterization of source European, American and, more importantly, genetically diverse African populations that contributed to the admixed pool of the Americas would enhance the present analysis.

## Supporting Information

File S1 mtDNA sequence data, NRY marker data and ethnic information.
(0.14 MB XLS)

Table S1 Primers used for sequencing mtDNA HVS I and II. Sequence pairs and the annealing temperatures used for amplification and sequencing of HVS I and HVS II regions [1].
(0.03 MB DOC)

Table S2 List of mtDNA RFLP assays. RFLP assays of mtDNA coding region that were used to ascertain the correct placement into a particular mtDNA haplogroup.
(0.05 MB DOC)

Table S3 List of SNPs typed for NRY. From left to right: SNP, nomenclature published by YCC in 2002 [2], nomenclature published by YCC in 2008 [3], SNP rs number. Rs numbers ending with the symbol # were typed using multiplex fragment analysis. In case rs numbers are not established yet, these SNPs can be typed using details in Hammer 1998 [4] (*), Underhill 2001 [5] (**), or Hammer 2001 [6] (***).
(0.06 MB DOC)

Table S4 Autosomal AIMs ancestry estimates. Estimated admixture for subset of African American (n = 31) and European American (n = 6) samples genotyped using commercially available Illumina ancestry panel of autosomal ancestry informative markers (AIMs). Estimates are reported by STRUCTURE software as average estimated membership in African, European and SE Asian/Native American clusters and average span of 90% probability interval (PI) that can be transposed to pseudo-standard error (pSE) by pSE = 1/2 (PI/1.645). Posterior probabilities (Ln P(D)) for K = 1–5 were the following: −731,387 (K1), −491,546 (K2), −469,725 (K3), −467,914 (K4), −466,163 (K5).
(0.03 MB DOC)

Table S5 MtDNA and NRY ancestry of populations from Philadelphia, Brazil and Cuba. Proportions of African, European, and Native American ancestry in the populations of Philadelphia, Brazil and Cuba of primarily African, European or mixed origin. (* This residual estimate is most likely influenced by the close genetic distance between SE Asian and Native American mtDNAs)
(0.05 MB DOC)

Table S6 MtDNA and NRY ancestry of general populations. Left: Proportions of African, European and Native American ancestry in the general populations of Cuba, Puerto Rico representing the Caribbean (with Cuba having 10% and Puerto Rico 7% of African population), Colombia, Brazil and Uruguay. Right: percentage by which African females (mtDNA) and males (NRY) contributed to the general population when only African Americans are considered as the only carriers of African mtDNAs and NRY, showing that all mtDNA estimates are >2-fold lower than the actual contribution of African mtDNAs to the whole population. (Note: *Afro-Caribbeans are from the islands of: Dominica, Grenada, Jamaica, St. Kitts, St. Lucia, St. Thomas, St. Vincent, and Trinidad and have much higher % of African populations compared to Cuba or Puerto Rico (e.g. Jamaica: 91% vs. Cuba/Puerto Rico 10% and 7%). Since there are no complementary data from general and Afro-Caribbean populations available, we assumed the admixture within African-derived populations being on average similar for the whole Caribbean and for calculations including Cuba/Puerto Rico we used the estimated admixture rates in African-derived populations for the whole Caribbean and demographic/genetic profiles of Cuban/Puerto Rican populations. Therefore, these estimates may be inaccurate.)
(0.10 MB PDF)

Table S7 Estimated mtDNA ancestry of “White” populations. The estimated proportions of African, European and Native American female ancestry to the pool of “White” Americans, based on the demographic data combined with mtDNA ancestry of African Americans, with a few exceptions (designated by *) where it was the mtDNA genetic variation of European Americans (Philadelphia = USA) and White Brazilians that was the sole source for calculating listed admixture estimates.
(0.03 MB DOC)

References S1 Supplementary references
(0.03 MB DOC)
