# Supplementary material for: Evaluation of Group Genetic Ancestry of Populations from Philadelphia and Dakar in the Context of Sex-Biased Admixture in the Americas
Source: PLoS One. 2009 Nov 25;4(11):e7842. doi: 10.1371/journal.pone.0007842 (PMC2776971; doi:10.1371/journal.pone.0007842)
Supplement: Table S1 — Primers used for sequencing mtDNA HVS I and II. Sequence pairs and the annealing temperatures used for amplification and sequencing of HVS I and HVS II regions [1]. (0.03 MB DOC) [file pone.0007842.s002.doc]

|  | Amplification PCR (Annealing Temp.) | Sequencing PCR |
| --- | --- | --- |
| HVS I | 15,838F: CCT-AAT-ACC-AAC-TAT-CTC-CC  269R: GGA-AAG-TGG-CTG-TGC-AGA-C  (53 °C) | 15,977F: CCA-CCA-TTA-GCA-CCC-AAA-GC  16,501R: GAT-GTC-GGA-TAC-AGT-TCA-C  (51 °C) |
| HVS II | 16,527F: CCT-AAA-TAG-CCC-ACA-CGT-TC  725R: GGT-GAA-CTC-ACT-GGA-ACG-GG  (55 °C) | 1F: GAT-CAC-AGG-TCT-ATC-ACC-CT  429R: CTG-TTA-AAA-GTG-CAT-ACC-GCC  (55 °C) and 725R |
