# Supplementary material for: Evaluation of Group Genetic Ancestry of Populations from Philadelphia and Dakar in the Context of Sex-Biased Admixture in the Americas
Source: PLoS One. 2009 Nov 25;4(11):e7842. doi: 10.1371/journal.pone.0007842 (PMC2776971; doi:10.1371/journal.pone.0007842)
Supplement: Table S2 — List of mtDNA RFLP assays. RFLP assays of mtDNA coding region that were used to ascertain the correct placement into a particular mtDNA haplogroup. (0.05 MB DOC) [file pone.0007842.s003.doc]

| Primarily of which continent | Haplogroup | Primer pair (5’-3’) | T_m_ (°C) | Primary location/Restr. enzyme cut position |
| --- | --- | --- | --- | --- |
| Asia/America | A | 251-269/725-706 | 55 | 663/ sequencing |
| Asia/America | B | 8188-8207/8366-8345 | 59 | 9-bp del./ sequencing |
| Asia/America | D | 5151-5170/5481-5464 | 45 | 5178A/ -Alu I 5176 |
| Asia | E | 7367-7384/7628-7610 | 49 | 7598/ -HhaI 7598 |
| Asia | G | 4651-4670/4952-4934 | 49 | 4833/ +HaeII 4830 |
| West Eurasia | H | 6890-6909/7131-7115 | 47 | 7028/ -AluI 7025 |
| West Eurasia | HV | 14407-14424/14810-14791 | 51 | 14766/ -MseI 14766 |
| West Eurasia | I | 4500-4519/4678-4659 | 51 | 4529T/ -HaeII 4529 |
| West Eurasia | J | 13537-13556/13851-13832 | 51 | 13708/ -BstNI 13704 |
| Africa | L0/1/2 | 3388-3409/3745-3725 | 53 | 3594/ +HpaI 3592 |
| Africa | L0/1 | 534-556/1189-1166 | 59 | 825A/ sequencing |
| Africa | L1 | 6890-6910/7339-7319 | 47 | 7055/ -AluI 7055 |
| Africa | L1b/L3e | 2200-2220/2458-2439 | 49 | 2352/ +MboI 2349 |
| Africa | L2a | 13537-13556/13851-13832 | 51 | 13803/ +HaeIII 13803 |
| Africa | L2b | 3951-3965/4325-4303 | 51 | 4158/ +AluI 4157 |
| Africa | L2c | 13809-13837/14148-14139 | 51 | 13958C/ -HaeIII 13957 |
| Africa | L2d | 3388-3409/3745-3725 | 53 | 3693/ -MboI 3693 |
| Africa | L3b | 9913-9931/10311-10293 | 49 | 10086/ +TaqI 10084 |
| Africa | L3d | 8566-8584/8880-8862 | 53 | 8618/ -MboI 8616 |
| Africa | L3h | 3951-3965/4678-4659 | 51 | 4388/ sequencing |
| Africa | L3f | 14407-14424/14810-14791 | 51 | 14769/ sequencing |
| Africa | L0/1/2/4 | 534-556/1189-1166 | 59 | 769/ sequencing |
| Asia | M | 10279-10296/10569-10550 | 51 | 10400/ +AluI 10397 (once 10398A->G) |
| West Eurasia | N (except I, N1a, J, K) | 10279-10296/10569-10550 | 51 | 10398/ -DdeI 10394 |
| West Eurasia | R | 12599-12618/12785-12766 | 51 | 12705/ +MboII 12705 |
| West Eurasia | T | 15409-15428/15720-15701 | 51 | 15607/ +AluI 15607 |
| West Eurasia | U+K | 12104-12124/12338-12309 | 53 | 12308/ +HinfI 12308 |
| West Eurasia | V | 4500-4519/4678-4659 | 51 | 4580/ -NlaII 4580 |
| West Eurasia | W | 8925-8953/9100-9081 | 53 | 8994/ -HaeIII 8994 |
| Asia/America | X | 14407-14424/14810-14791 | 51 | 14465/ +AccI 14464 |
