# Supplementary material for: Evaluation of Group Genetic Ancestry of Populations from Philadelphia and Dakar in the Context of Sex-Biased Admixture in the Americas
Source: PLoS One. 2009 Nov 25;4(11):e7842. doi: 10.1371/journal.pone.0007842 (PMC2776971; doi:10.1371/journal.pone.0007842)
Supplement: Table S3 — List of SNPs typed for NRY. From left to right: SNP, nomenclature published by YCC in 2002 [2], nomenclature published by YCC in 2008 [3], SNP rs number. Rs numbers ending with the symbol # were typed using multiplex fragment analysis. In case rs numbers are not established yet, these SNPs can be typed using details in Hammer 1998 [4] (*), Underhill 2001 [5] (**), or Hammer 2001 [6] (***). (0.06 MB DOC) [file pone.0007842.s004.doc]

|  | YCC 2002 | **YCC 2008** | rs number |
| --- | --- | --- | --- |
| M91 | A | **A** | rs2032651# |
| M60 | B | **B** | rs2032623# |
| M168 | C-R | **C-R** | rs2032595 |
| M216 | C | **C** | rs2032666 |
| M174 | D | **D** | rs2032602 |
| M96 | E | **E** | rs9306841 |
| M89 | F-R | **F-R** | rs2032652 |
| M201 | G | **G** | rs2032636 |
| M69 | H | **H** | rs2032673 |
| M9 | K-R | **K-R** | rs3900 |
| M170 | I | **I** | rs2032597 |
| M304 |  | **J** | rs13447352 |
| M20 | L | **L** | rs3911 |
| M214 | O | **NO** | rs2032674 |
| M231 | N | **N** | rs9341278 |
| M175 | O | **O** | rs2032678 |
| P31 | O2 | **O2** | no rs*** |
| M45 | P | **P** | rs2032631 |
| M242 |  | **Q** | rs8179021 |
| M207 | R | **R** | rs2032658 |
| M132 (M33) | E1 | **E1a** | rs2032617 |
| M75 | E2 | **E2** | rs2032639 |
| PN2 | E3 | **E1b1** | no rs* |
| M2 | E3a | **E1b1a** | rs3893 |
| M191 | E3a7 | **E1b1a7** | rs2032590 |
| M35 | E3b | **E1b1b1** | no rs** |
| M148 | E3b1a | **E1b1b1a3a** | no rs** |
| M81 | E3b2 | **E1b1b1a** | rs2032640 |
| M123 | E3b3 | **E1b1b1c** | no rs** |
| M267 | J1 | **J1** | rs9341313 |
| M172 | J2 | **J2** | rs2032604 |
| M12 | J2e | **J2b** | rs3903 |
| M223 | I1 | **I2b** | no rs** |
| M173 | R1 | **R1** | rs2032624 |
| M124 | R2 | **R2** | no rs** |
| M178 | N3a | **N1c1** | no rs** |
| M102 | J2e1 | **J2b** | rs2032608 |
| P25 | R1b | **R1b1** | rs150173 |
| M269 | R1b3 | **R1b1b2** | rs9786153 |
| M120 | Q1 | **Q1a1** | no rs** |
| M25 | Q2 | **Q1a2** | no rs** |
| M3 | Q3 | **Q1a3a** | rs3894 |
