# Supplementary material for: Evaluation of Group Genetic Ancestry of Populations from Philadelphia and Dakar in the Context of Sex-Biased Admixture in the Americas
Source: PLoS One. 2009 Nov 25;4(11):e7842. doi: 10.1371/journal.pone.0007842 (PMC2776971; doi:10.1371/journal.pone.0007842)
Supplement: Table S4 — Autosomal AIMs ancestry estimates. Estimated admixture for subset of African American (n = 31) and European American (n = 6) samples genotyped using commercially available Illumina ancestry panel of autosomal ancestry informative markers (AIMs). Estimates are reported by STRUCTURE software as average estimated membership in African, European and SE Asian/Native American clusters and average span of 90% probability interval (PI) that can be transposed to pseudo-standard error (pSE) by pSE = 1/2 (PI/1.645). Posterior probabilities (Ln P(D)) for K = 1–5 were the following: −731,387 (K1), −491,546 (K2), −469,725 (K3), −467,914 (K4), −466,163 (K5). (0.03 MB DOC) [file pone.0007842.s005.doc]

| Estimates for K=3 | African (± pSE/PI) | European (± pSE/PI) | SE Asian/Nat.Am. (± pSE/PI) |
| --- | --- | --- | --- |
| African American (n=31) | 74.4% (± 1.3/4.3) | 23.7% (± 2.2/7.1) | 1.9% (± 2.1/6.9) |
| European American (n=6) | 2.5% (± 0.9/3) | 95.7% (± 1.9/6.3) | 1.9% (± 2/6.5) |
