# Supplementary material for: Evaluation of Group Genetic Ancestry of Populations from Philadelphia and Dakar in the Context of Sex-Biased Admixture in the Americas
Source: PLoS One. 2009 Nov 25;4(11):e7842. doi: 10.1371/journal.pone.0007842 (PMC2776971; doi:10.1371/journal.pone.0007842)
Supplement: Table S5 — MtDNA and NRY ancestry of populations from Philadelphia, Brazil and Cuba. Proportions of African, European, and Native American ancestry in the populations of Philadelphia, Brazil and Cuba of primarily African, European or mixed origin. (* This residual estimate is most likely influenced by the close genetic distance between SE Asian and Native American mtDNAs) (0.05 MB DOC) [file pone.0007842.s006.doc]

|  |  | **mtDNA** | n=819 | n=3532 | n=57 | **NRY** | n=834 | n=481 | n=398 |
| --- | --- | --- | --- | --- | --- | --- | --- | --- | --- |
| Population | Geography | n | W Africa | Europe | America | n | W Africa | Europe | America |
| *African American* | **Philadelphia** | **217** | **89.2% ± 3** | **9.1% ± 3** | **1.7% ± 0.9** | **199** | **67.5% ± 4** | **31.2% ± 4** | **1.3% ± 1.5** |
|  | **Brazil [7,8,9,10]** | **277** | **84.1% ± 3** | **1.7% ± 1** | **14.2% ± 3** | **380** | **51.2% ± 3** | **48.1% ± 3** | 0.7% ± 1.2 |
| *European American* | **Philadelphia** | **204** | 5.4% ± 5 | **93% ± 7** | 1.6% ± 2^*^ | **190** | 0.5% ± 1.4 | **98.3% ± 3** | **1.2% ± 1.5** |
|  | **Brazil [8,11]** | **247** | **25.6% ± 5** | **49.2% ± 4** | **25.2% ± 4** | **180** | **0** | **100% ± 3** | **0** |
| *Mixed* | **Cuba [12]** | **245** | **52.4%± 7** | **19.7% ± 4** | **27.9% ± 6** | **132** | **13.4%± 3** | **86.4% ± 4** | 0.2% ± 1 |
