# Supplementary material for: Evaluation of Group Genetic Ancestry of Populations from Philadelphia and Dakar in the Context of Sex-Biased Admixture in the Americas
Source: PLoS One. 2009 Nov 25;4(11):e7842. doi: 10.1371/journal.pone.0007842 (PMC2776971; doi:10.1371/journal.pone.0007842)
Supplement: Table S6 — MtDNA and NRY ancestry of general populations. Left: Proportions of African, European and Native American ancestry in the general populations of Cuba, Puerto Rico representing the Caribbean (with Cuba having 10% and Puerto Rico 7% of African population), Colombia, Brazil and Uruguay. Right: percentage by which African females (mtDNA) and males (NRY) contributed to the general population when only African Americans are considered as the only carriers of African mtDNAs and NRY, showing that all mtDNA estimates are >2-fold lower than the actual contribution of African mtDNAs to the whole population. (Note: *Afro-Caribbeans are from the islands of: Dominica, Grenada, Jamaica, St. Kitts, St. Lucia, St. Thomas, St. Vincent, and Trinidad and have much higher % of African populations compared to Cuba or Puerto Rico (e.g. Jamaica: 91% vs. Cuba/Puerto Rico 10% and 7%). Since there are no complementary data from general and Afro-Caribbean populations available, we assumed the admixture within African-derived populations being on average similar for the whole Caribbean and for calculations including Cuba/Puerto Rico we used the estimated admixture rates in African-derived populations for the whole Caribbean and demographic/genetic profiles of Cuban/Puerto Rican populations. Therefore, these estimates may be inaccurate.) (0.10 MB PDF) [file pone.0007842.s007.pdf]

General population	mtDNA			NRY			% of African lineages in general population contributed by African-derived populations			% of total pop
	Africa	Europe	America	Africa	Europe	America	AA of	mtDNA	NRY	
USA	*NA*	NA	NA	*NA*	NA	NA	USA	*12.3%*	*9.6%*	*13%*
Cuba [12]	*52.3%*	19.7%	27.9%	*13.5%*	86.5%	0%	Caribbean	*9.0%*	*6.8%*	*10%**
Puerto Rico [13]	*27.2%*	11.5%	61.3%	*NA*	NA	NA				
Colombia [14]	*8%*	2%	90%	*5%*	94%	1%	Colombia	*3.2%*	*2.5%*	*4%*
Brazil [15,16,17]	*23.7%*	37%	39.5%	*4%*	92.2%	2.7%	Brazil	*5.4%*	*3.7%*	*7%*
Uruguay [18,19]	*18.9%*	34.9%	46.3%	*1.6%*	96.5%	1.9%	Uruguay	*2.1%*	*1.2%*	*4%*

>2-fold difference
