# Supplementary material for: Evaluation of Group Genetic Ancestry of Populations from Philadelphia and Dakar in the Context of Sex-Biased Admixture in the Americas
Source: PLoS One. 2009 Nov 25;4(11):e7842. doi: 10.1371/journal.pone.0007842 (PMC2776971; doi:10.1371/journal.pone.0007842)
Supplement: Table S7 — Estimated mtDNA ancestry of “White” populations. The estimated proportions of African, European and Native American female ancestry to the pool of “White” Americans, based on the demographic data combined with mtDNA ancestry of African Americans, with a few exceptions (designated by *) where it was the mtDNA genetic variation of European Americans (Philadelphia = USA) and White Brazilians that was the sole source for calculating listed admixture estimates. (0.03 MB DOC) [file pone.0007842.s008.doc]

| **White** | **% of total pop** | **mtDNA** | | |
| --- | --- | --- | --- | --- |
|  |  | Af | **Eu** | NAm |
| **USA*** | 80% | 6% | **93%** | 1% |
| **Cuba**  **Puerto Rico** | 65% | 45% | **30%** | 25% |
|  | 76% | 15% | **15%** | 70% |
| **Colombia** | 20% | 0% | **10%** | 90% |
| **Brazil*** | 50% | 30% | **40%** | 30% |
| **Uruguay** | 88% | 15% | **40%** | 45% |
