# Supplementary material for: Evaluation of Group Genetic Ancestry of Populations from Philadelphia and Dakar in the Context of Sex-Biased Admixture in the Americas
Source: PLoS One. 2009 Nov 25;4(11):e7842. doi: 10.1371/journal.pone.0007842 (PMC2776971; doi:10.1371/journal.pone.0007842)
Supplement: References S1 — Supplementary references (0.03 MB DOC) [file pone.0007842.s009.doc]

**Supplementary References:**

1. Gokcumen O, Dulik MC, Pai AA, Zhadanov SI, Rubinstein S, et al. (2008) Genetic variation in the enigmatic Altaian Kazakhs of South-Central Russia: insights into Turkic population history. Am J Phys Anthropol 136: 278-293.

2. Y-Chromosome-Consortium (2002) A nomenclature system for the tree of human Y-chromosomal binary haplogroups. Genome Res 12: 339-348.

3. Karafet TM, Mendez FL, Meilerman MB, Underhill PA, Zegura SL, et al. (2008) New binary polymorphisms reshape and increase resolution of the human Y chromosomal haplogroup tree. Genome Res 18: 830-838.

4. Hammer MF, Karafet T, Rasanayagam A, Wood ET, Altheide TK, et al. (1998) Out of Africa and back again: nested cladistic analysis of human Y chromosome variation. Mol Biol Evol 15: 427-441.

5. Underhill PA, Kivisild T (2007) Use of y chromosome and mitochondrial DNA population structure in tracing human migrations. Annu Rev Genet 41: 539-564.

6. Hammer MF, Karafet TM, Redd AJ, Jarjanazi H, Santachiara-Benerecetti S, et al. (2001) Hierarchical patterns of global human Y-chromosome diversity. Mol Biol Evol 18: 1189-1203.

7. Goncalves VF, Carvalho CM, Bortolini MC, Bydlowski SP, Pena SD (2008) The phylogeography of African Brazilians. Hum Hered 65: 23-32.

8. Guerreiro V, Bisso-Machado R, Marrero A, Hunemeier T, Salzano FM, et al. (2009) Genetic signatures of parental contribution in black and white populations in Brazil. Genetics and Molecular Biology 32: 1-11.

9. Hunemeier T, Carvalho C, Marrero AR, Salzano FM, Junho Pena SD, et al. (2007) Niger-Congo speaking populations and the formation of the Brazilian gene pool: mtDNA and Y-chromosome data. Am J Phys Anthropol 133: 854-867.

10. Silva WA, Bortolini MC, Schneider MP, Marrero A, Elion J, et al. (2006) MtDNA haplogroup analysis of black Brazilian and sub-Saharan populations: implications for the Atlantic slave trade. Hum Biol 78: 29-41.

11. Alves-Silva J, da Silva Santos M, Guimaraes PE, Ferreira AC, Bandelt HJ, et al. (2000) The ancestry of Brazilian mtDNA lineages. Am J Hum Genet 67: 444-461.

12. Mendizabal I, Sandoval K, Berniell-Lee G, Calafell F, Salas A, et al. (2008) Genetic origin, admixture, and asymmetry in maternal and paternal human lineages in Cuba. BMC Evol Biol 8: 213.

13. Martinez-Cruzado JC, Toro-Labrador G, Viera-Vera J, Rivera-Vega MY, Startek J, et al. (2005) Reconstructing the population history of Puerto Rico by means of mtDNA phylogeographic analysis. Am J Phys Anthropol 128: 131-155.

14. Carvajal-Carmona LG, Soto ID, Pineda N, Ortiz-Barrientos D, Duque C, et al. (2000) Strong Amerind/white sex bias and a possible Sephardic contribution among the founders of a population in northwest Colombia. Am J Hum Genet 67: 1287-1295.

15. Barbosa AB, da Silva LA, Azevedo DA, Balbino VQ, Mauricio-da-Silva L (2008) Mitochondrial DNA control region polymorphism in the population of Alagoas state, north-eastern Brazil. J Forensic Sci 53: 142-146.

16. Batista dos Santos SE, Rodrigues JD, Ribeiro-dos-Santos AK, Zago MA (1999) Differential contribution of indigenous men and women to the formation of an urban population in the Amazon region as revealed by mtDNA and Y-DNA. Am J Phys Anthropol 109: 175-180.

17. Silva DA, Carvalho E, Costa G, Tavares L, Amorim A, et al. (2006) Y-chromosome genetic variation in Rio de Janeiro population. Am J Hum Biol 18: 829-837.

18. Bonilla C, Bertoni B, Gonzalez S, Cardoso H, Brum-Zorrilla N, et al. (2004) Substantial Native American female contribution to the population of Tacuarembo, Uruguay, reveals past episodes of sex-biased gene flow. Am J Hum Biol 16: 289-297.

19. Sans M, Weimer TA, Franco MH, Salzano FM, Bentancor N, et al. (2002) Unequal contributions of male and female gene pools from parental populations in the African descendants of the city of Melo, Uruguay. Am J Phys Anthropol 118: 33-44.
